# α-synuclein inclusions are abundant in non-neuronal cells in the anterior olfactory nucleus of the Parkinson’s disease olfactory bulb

**DOI:** 10.1038/s41598-020-63412-x

**Published:** 2020-04-21

**Authors:** Taylor J. Stevenson, Helen C. Murray, Clinton Turner, Richard L. M. Faull, Birger V. Dieriks, Maurice A. Curtis

**Affiliations:** 1 Department of Anatomy and Medical Imaging, University of Auckland, Auckland, New Zealand; 2 Centre for Brain Research, University of Auckland, Auckland, New Zealand; 3 Deparment of Anatomical Pathology, LabPlus, Auckland City Hospital, Auckland, New Zealand

**Keywords:** Diseases of the nervous system, Parkinson's disease, Neuroscience, Medical research

## Abstract

Reduced olfactory function (hyposmia) is one of the most common non-motor symptoms experienced by those living with Parkinson’s disease (PD), however, the underlying pathology of the dysfunction is unclear. Recent evidence indicates that α-synuclein (α-syn) pathology accumulates in the anterior olfactory nucleus of the olfactory bulb years before the motor symptoms are present. It is well established that neuronal cells in the olfactory bulb are affected by α-syn, but the involvement of other non-neuronal cell types is unknown. The occurrence of intracellular α-syn inclusions were quantified in four non-neuronal cell types – microglia, pericytes, astrocytes and oligodendrocytes as well as neurons in the anterior olfactory nucleus of post-mortem human PD olfactory bulbs (n = 11) and normal olfactory bulbs (n = 11). In the anterior olfactory nucleus, α-syn inclusions were confirmed to be intracellular in three of the four non-neuronal cell types, where 7.78% of microglia, 3.14% of pericytes and 1.97% of astrocytes were affected. Neurons containing α-syn inclusions comprised 8.60% of the total neuron population. Oligodendrocytes did not contain α-syn. The data provides evidence that non-neuronal cells in the PD olfactory bulb contain α-syn inclusions, suggesting that they may play an important role in the progression of PD.

## Introduction

Parkinson’s disease (PD) is clinically diagnosed by the presence of four cardinal motor symptoms: bradykinesia, tremor, rigidity and postural instability. The motor symptoms are caused by the loss of dopaminergic neurons in the substantia nigra pars compacta^1^. However, non-motor symptoms can precede motor symptoms by more than a decade^2^ and include olfactory dysfunction, rapid eye movement sleep behaviour disorder and autonomic disorders such as constipation. The non-motor symptoms and motor symptoms appear to coincide with the aggregation of the pathological protein α-synuclein (α-syn) in specific brain regions^2–4^. Phosphorylated α-syn pathology affects different brain regions in a sequential pattern that has been characterised into six stages^5^. In stage I, the dorsal motor nucleus of the vagus nerve and the olfactory system – olfactory mucosa, olfactory bulb (OFB) and regions of the anterior olfactory nucleus (AON) present α-syn pathology. As the disease progresses into stage II, α-syn pathology is evident in the brainstem where it reaches the substantia nigra in stage III, coinciding with the clinical motor symptoms associated with PD^5^. As the disease continues through stages IV – VI, α-syn begins to affect cortical regions.

**Figure 1:**
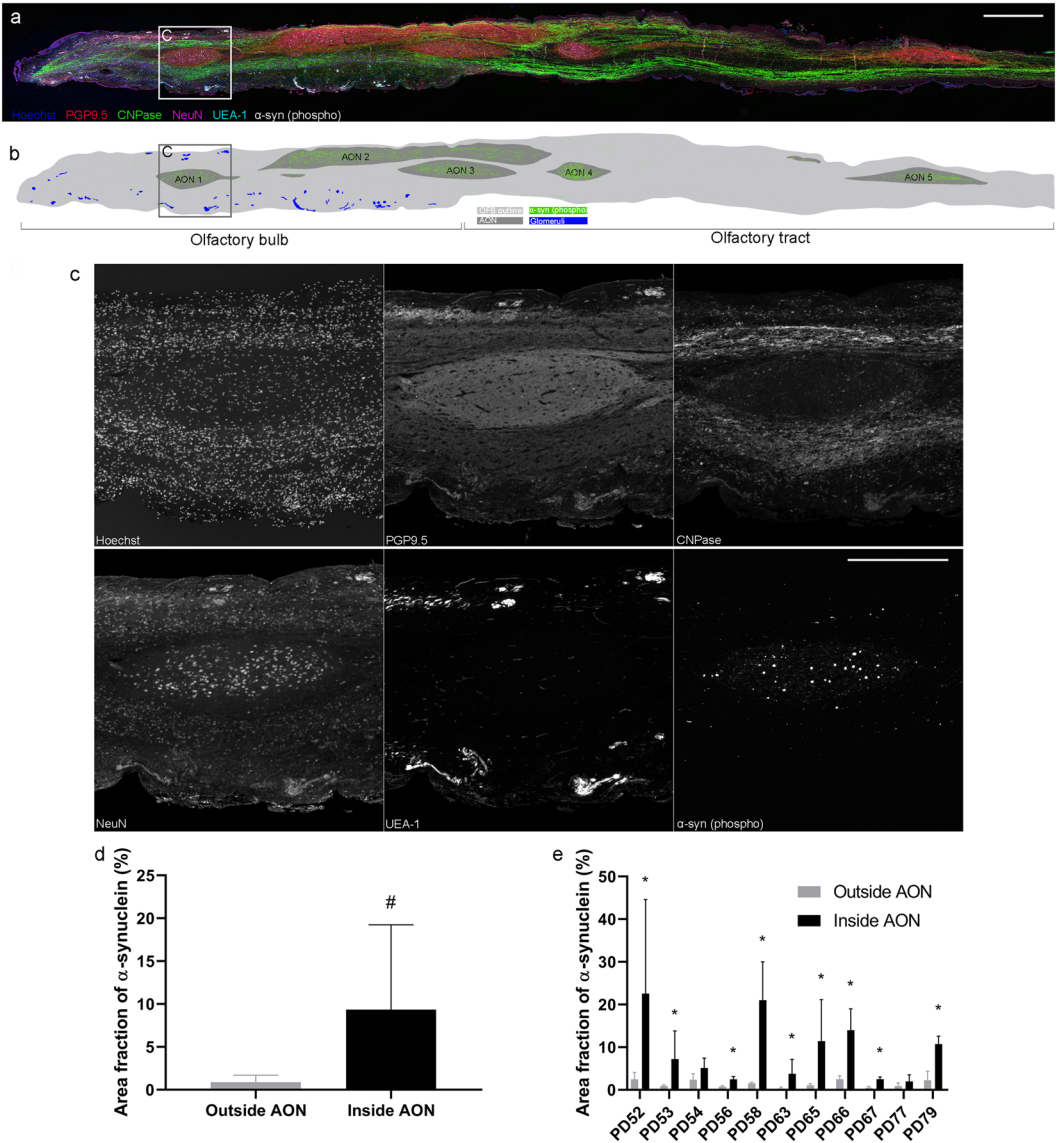
(**a**) Immunofluorescence image of a sagittal section from a human PD OFB stained with antibodies for PGP9.5, CNPase, NeuN, UEA-1, and a Hoechst counterstain to identify the AON regions. Phosphorylated α-syn (s129) antibody was used for the distribution of α-syn. (**b**) Illustration of the PD OFB and tract displaying the distribution of α-syn, highlighting that most of the α-syn inclusions are in the multiple AON regions. (**c**) Inset of AON 1 showing representative immunofluorescence images used to identify the AON region. The AON is characterized by a decreased density of cells represented with Hoechst staining, increased PGP9.5 immunoreactivity, a lack of CNPase immunoreactivity and increased density of large NeuN-positive cells. In PD OFBs, the increased abundance of α-syn can be used to identify the AON. Scale bar, 1 mm (**a**) and 500 µm (**c**). Graphs showing (**d**) area fraction of α-syn (%) inside versus outside of the AON of human PD OFBs (n = 11) and (**e**) area fraction of α-syn (%) inside versus outside of the AON by case. Data presented as mean ± SD. *# p* < 0.0001, * p < *0.05*.

One of the most common non-motor symptoms that is present in more than 90% of those suffering with PD is the loss of olfaction (hyposmia or anosmia depending on severity)^6^. Prior to the changes seen in the substantia nigra, α-syn pathology is present throughout the OFB and tract and is especially abundant in each compartment of the AON^5,7,8^. The AON is the neural conduit between the OFB and the piriform cortex, entorhinal cortex, amygdala and hippocampal formations^9,10^. The olfactory system consists of olfactory mucosa in the roof of the nose that houses the olfactory sensory neurons whose axons traverse the cribriform plate and form the olfactory nerve layer of the OFB. Evidence from studies of the rodent OFB structure suggests that glomeruli are formed from olfactory sensory neuron fibres and then project to mitral cells and mitral cells synapse in the AON. Thus, the olfactory sensory neurons, which are exposed to the mucus and external environment, are just one synapse between the external environment and the AON (Fig. 1a)^11,12^. In a theory called the olfactory vector hypothesis, it is proposed that the close connectional proximity of the external environment to the central brain structures allows inhaled environmental toxins and pathogens to enter the brain via the AON^6,11,12^.

The spread of α-syn from the OFB to deeper brain regions is evidenced by a previous study showing that injection of α-syn inclusions in the OFB of mice results in α-syn-positive inclusions developing in many interconnected brain regions over 12 months^13,14^. Local OFB interneurons in the mouse and human AON are also particularly good at internalising α-syn^13,15,16^. Indeed many *in vitro* and *in vivo* models have suggested that neuronal cells are the main facilitator for the spread of α-syn pathology following the major neuronal pathways in the brain^17–19^. These studies point toward the AON and OFB as being crucial structures for the spread of α-syn pathology. But what of other cell types that may spread, but may not aggregate α-syn?

The involvement of non-neuronal cells in the spread of α-syn pathology in the PD brain has been overlooked. Microglia and astrocytes are predominantly associated with inflammatory processes in the PD brain^20–25^. However, recent *in vitro* studies demonstrate that microglia and astrocytes efficiently take up and degrade α-syn from extracellular locations^26,27^. In the human PD brain, the number of astrocytes and oligodendrocytes containing α-syn inclusions appear to correlate with the severity of nigral neuronal loss^28,29^. Most recently, *in vitro* evidence suggests that pericytes, a blood-vessel associated cell involved in the maintenance of the blood brain barrier, together with astrocytes may be involved in the spread of α-syn from one cell to the next^30–32^.

Taken together, current literature suggests that non-neuronal cells could play an active role in the progression of PD, but evidence of these cells containing α-syn in the human PD OFB is lacking. Here, we show that in the PD AON, α-syn is found within neurons, microglia, pericytes and astrocytes but not oligodendrocytes. Secondly, we observed that the α-syn structures in non-neuronal cells look similar to some of the α-syn inclusions seen in neuronal cells, suggesting that non-neuronal cells may play a more active role in the pathogenesis of PD than previously thought.

## Results

### Distribution of phosphorylated α-synuclein in the human Parkinson’s disease olfactory bulb

Phosphorylated α-syn inclusions were present throughout the OFB and tract of the 11 PD cases used in this study (Fig. 1a–c). Of the 11 normal cases, only OFB55 contained phosphorylated α-syn in the glomerular layer of the OFB in small amounts. In the PD OFBs, phosphorylated α-syn-positive Lewy neurites and Lewy bodies were seen in the glomerular layer, external plexiform, mitral cell layer, internal plexiform layer, granule cell layers and in the multiple AON compartments. To confidently identify the AON regions across different sections and cases, we found that the co-labelling of Hoechst, NeuN, PGP9.5 and CNPase was sufficient. The AON has a reduced number of Hoechst positive cells, clusters of large NeuN positive neurons and increased PGP9.5 immunoreactivity. Regarding myelination, there is a lack of CNPase immunoreactivity in the AON but positive immunoreactivity labelling the myelinated fiber tracts in the acellular neuropil zone creating a definitive border around the AON. Lastly, in PD OFBs, increased abundance of phosphorylated α-syn staining can be seen (Fig. 1a,c).

The area fraction of α-syn was significantly more abundant in the AON regions covering 9.35% ± 9.88% of the surface area compared with the area fraction of α-syn outside of the AON only covering 0.87% ± 0.81% of the surface area in the 11 PD cases (p < 0.0001; Fig. 1d). All 11 PD OFB cases had phosphorylated α-syn labelling in the AON, however, the amount of α-syn present varied greatly between cases (0.36–2.52% outside AON, 1.98–22.55% inside AON; Fig. 1e). As the majority of α-syn was found within the AON regions in the PD OFBs (Fig. 1d), quantification of the cells that contained intracellular α-syn were confined to this region. None of the normal OFBs had phosphorylated α-syn in the AON, therefore, the number of cells containing α-syn inclusions were zero.

**Figure 2:**
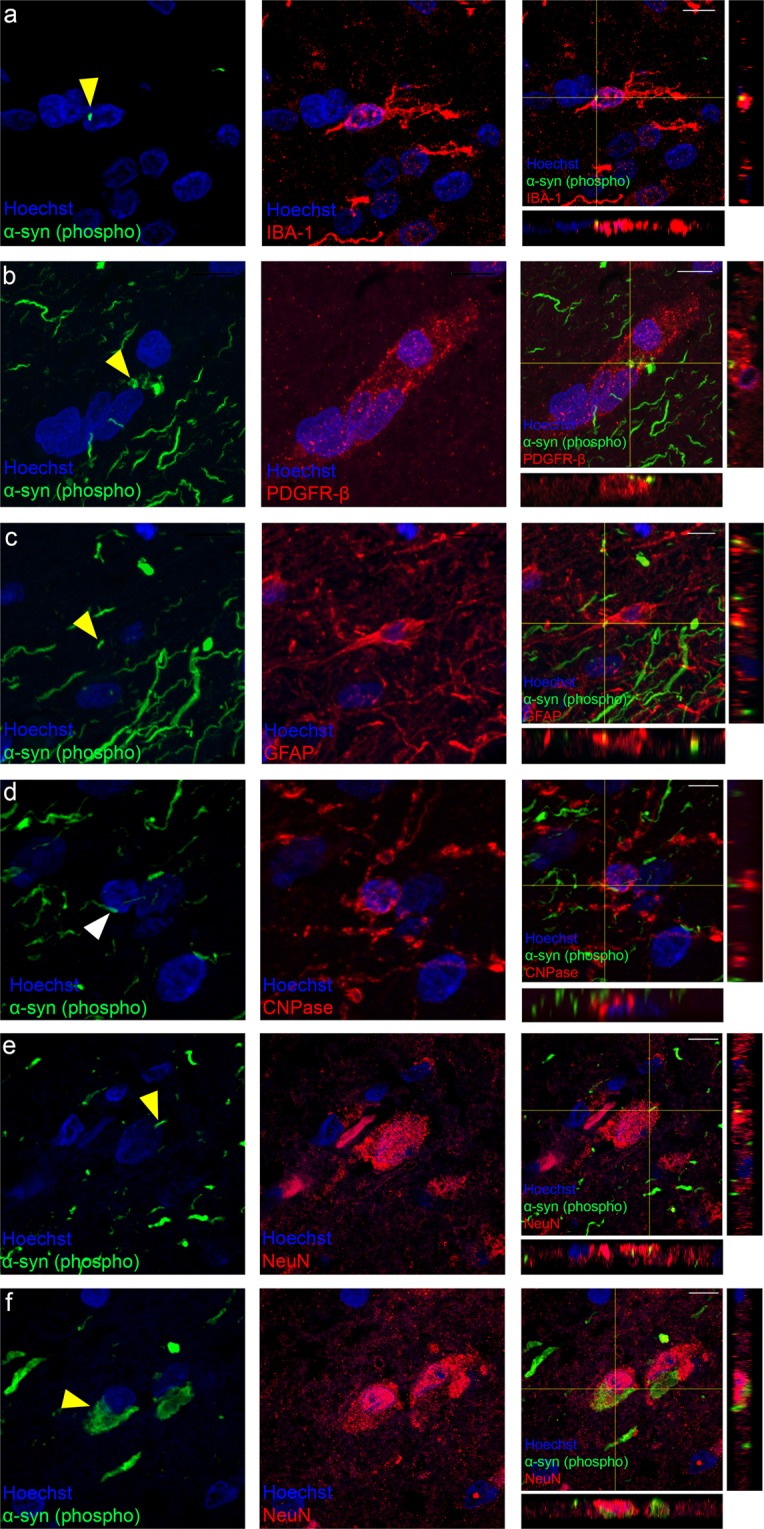
Representative confocal immunofluorescence images with orthogonal views of different cell types containing α-syn in the AON of human PD OFBs. Small intracellular α-syn inclusions were found in (**a**) microglia, (**b**) pericytes and (**c**) astrocytes. No intracellular α-syn was found in (**d**) oligodendrocytes. Neurons either contained (**e**) small α-syn inclusions or (**f**) large Lewy body like inclusions. Yellow arrows indicate α-syn inclusions that are intracellular. White arrows indicate α-syn inclusions that are extracellular. Scale bar, 10 µm.

### Quantification of the different cell types containing phosphorylated α-synuclein inclusions in the human Parkinson’s disease olfactory bulb

α-syn inclusions were present in microglia, pericytes and astrocytes but not in oligodendrocytes in the AON of the human PD OFB (Fig. 2a–d). We observed that these non-neuronal cells only contained small α-syn inclusions. However, neurons contained either small α-syn inclusions that resemble those seen in the non-neuronal cells (Fig. 2e) or the classical large Lewy body-like inclusions (Fig. 2f).

In this study, we used a two-step process to quantify cells containing α-syn. First, widefield tiled fluorescent images were used to identify cells that potentially contained intracellular α-syn. Cumulatively in the 11 PD cases, 783 microglia, 458 pericytes, 529 astrocytes and 80 oligodendrocytes were counted. In six PD cases, 495 neurons were identified that potentially contained α-syn. The presence of intracellular α-syn was difficult to quantify using simple co-localisation techniques. α-syn inclusions can appear in close proximity to cells of interest and co-localise with the specific cellular markers, however, it can be unclear whether the α-syn inclusions were intracellular, above or below the cell of interest (Supplementary Fig. 1a,b). To overcome this issue, we carried out a second quantification step by imaging each cell that potentially contained intracellular α-syn using confocal microscopy (Supplementary Fig. 1a,a’). Therefore, each cell that was identified to potentially contain α-syn was reimaged to confirm that the α-syn was intracellular (Supplementary Fig. 1b,b’). Following this, it was confirmed that 261 microglia, 89 pericytes, 119 astrocytes, zero oligodendrocytes and 182 neurons contained intracellular α-syn. Without the use of confocal microscopy, we would have overestimated the number of cells that contained intracellular α-syn by 3.5 times.

**Figure 3:**
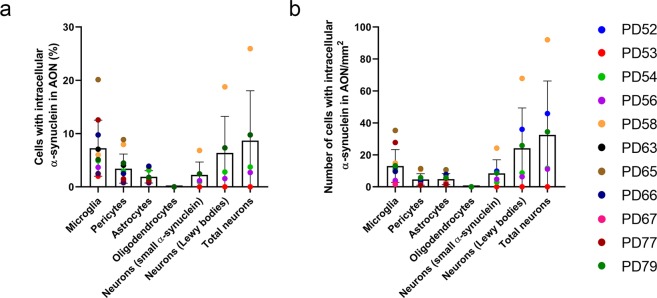
Graphs showing (**a**) Percentage of cells with intracellular α-syn in the AON of PD OFBs (n = 11), where, 7.78% ± 5.22% of microglia, 3.41% ± 2.74% of pericytes, 1.97% ± 1.17% of astrocytes and 0% of oligodendrocytes contained intracellular α-syn. Whereas, 2.25% ± 2.41% of neurons contained small α-syn inclusions, 6.38% ± 6.83% of neurons contained Lewy bodies, giving a total of 8.68% ± 9.34% α-syn containing neurons overall. (**b**) Number of cells with intracellular α-syn in the AON/mm^2^ (n = 11), microglia (14.02 ± 10.23 cells/mm^2^), astrocytes (5.09 ± 3.55 cells/mm^2^), pericytes (4.93 ± 3.60 cells/mm^2^) and oligodendrocytes (0 cells /mm^2^). Neurons either contained small α-syn inclusions (8.84 ± 8.56 cells/mm^2^) or Lewy bodies (24.10 ± 25.25 cells/mm^2^) comprising a total number of neurons (32.49 ± 33.69 cells/mm^2^) containing intracellular α-syn. Data presented as mean ± SD.

The total number of cells in each cell population was also counted in the AON regions of the OFB and tract to quantify the percentage of cells affected by α-syn. Cumulatively in the 11 PD cases, a total of 5288 microglia, 6925 astrocytes, 4419 pericytes,1621 oligodendrocytes and 2387 neurons were counted. Of the non-neuronal cell types, on average, 7.78% ± 5.22% of microglia, 3.41 ± 2.74% of pericytes and 1.97 ± 1.17% of astrocytes contained intracellular α-syn (Fig. 3a). A total of 12.56 ± 7.98% of non-neuronal cells were affected by α-syn. On average, there were 2.25 ± 2.41% of neurons that contained small α-syn inclusions, 6.38 ± 6.83% of neurons that contained Lewy body like inclusions and 8.68 ± 9.34% of the total neuronal population contained intracellular α-syn inclusions (Fig. 3a). There was a moderate positive correlation between the percentage of total cells containing intracellular α-syn in the AON and the area fraction of α-syn in the AON (r^2^ = 0.55; p = 0.08).

Additionally, the number of cells containing intracellular α-syn were normalised to the surface area of the AON. Of the non-neuronal cell types, on average, there were 14.02 ± 10.23 microglia/mm^2^, 4.93 ± 3.60 pericytes/mm^2^, and 5.09 ± 3.55 astrocytes/mm^2^ that contained intracellular α-syn inclusions (Fig. 3b). A total of 22.43 ± 14.44 non-neuronal cells/mm^2^ contained intracellular α-syn. On average, there were 8.84 ± 8.56 neurons with small α-syn/mm^2^, 24.10 ± 25.25 neurons with Lewy bodies/mm^2^ and a total of 32.49 ± 33.69 neurons/mm^2^ containing intracellular α-syn inclusions (Fig. 3b). There was a moderate positive correlation between the total number of cells/mm^2^ in the AON and the area fraction of α-syn in the AON (r^2^ = 0.64; p = 0.05).

Post-mortem human tissue often produces substantial variability that may be caused by a non-homogenous sample, where, interindividual variability, disease duration, age of death and post-mortem delay may all be contributing factors. We investigated this variability by looking for correlations between the total number of cells/mm^2^ containing α-syn compared to disease duration (R^2^ = 0.09, p = 0.54), age at death (R^2^ = 0.35, p = 0.21) and post-mortem delay (R^2^ = 0.006217, p = 0.88), however, no significant correlations were found.

## Discussion

Consistent with other studies, the majority of phosphorylated α-syn was present in the AON regions of the OFB and tract^5,16^, however, α-syn pathology was also present in all the other layers of the PD OFB (Fig. 1a,b). The human AON is a complex structure that has been poorly characterised. Previous descriptions of the AON have typically subdivided it into four regions^16^, however, more recently it has been suggested that the AON can be represented by 7 divisions along the rostro-caudal axis^33^. Nissl-stained sections demonstrate differential cellular density and morphology between the AON and the rest of the OFB. Clusters of medium to large sized pyramidal neurons with a diameter of 15–20 µm are often used to identify the AON region^7,16,34,35^. In PD cases, the dense accumulation of α-syn has previously been used to delineate the AON regions, however, this does not allow for comparison between cases that do not have a significant amount of phosphorylated α-syn. Previous characterisations of the AON made it difficult to remain consistent when identifying multiple AON segments. The size and location of the AON can vary between sections within the same OFB and therefore significantly between different OFB cases. It also remains unclear whether the different AON segments are connected or have functionally distinctive roles^36^. In this study, we used four cellular markers to target different structures of the OFB to more accurately delineate the AON, however, we did not make a distinction between the AON subdivisions (Fig. 1a,c).

The human AON is an area that is particularly affected by α-syn pathology early in the disease process^5,9^. Evidence from rodent studies suggest that the AON receives direct projections from the different structures in the OFB including ipsilateral and contralateral centrifugal projections that include projections back to the OFB structures. There are projections to many of the secondary olfactory structures such as the entorhinal cortices, tertiary projections to brain regions such as the periamygdaloid cortex and rostral entorhinal cortex as well as projections from at least 27 non-olfactory regions^34,37,38^. The structure of the OFB and the connections of the AON to the various other brain regions puts the AON in a position that could underlie the preferential involvement of α-syn pathology early in the disease. Previous studies highlight that neuronal cells such as mitral cells, neighbouring interneurons which express calcium-binding proteins, tyrosine hydroxylase expressing neurons and somatostatin neurons are affected by α-syn pathology and their projections to the tertiary structures deeper in the brain may provide a platform for the spread of α-syn along neural pathways^15,16,38–40^. In fact, *in vivo* studies in which various forms of α-syn inclusions have been injected into the OFB of mice have demonstrated that α-syn was detected in interconnected brain regions. These included the AON, frontal cortex, olfactory tubercle, periform cortex, striatum and amygdala. Local OFB interneurons were particularly good at internalising α-syn and it was likely that it would be propagating along these neural pathways into deeper brain regions^13^.

Supporting previous studies, this study demonstrates that neurons in the OFB are affected by α-syn pathology (Fig. 3a,b)^15,16^. However, we demonstrated that three of the four non-neuronal cells investigated – microglia, pericytes and astrocytes contained intracellular α-syn in the AON of the human PD OFB. Microglia are excellent phagocytes and are likely to be the main cell involved in the clearing of α-syn from the extracellular space^19,26,41^. *In vitro* evidence suggests that astrocytes can take up α-syn inclusions from the extracellular space and efficiently degrade it^19,26^. Animal studies that have injected α-syn into different brain regions such as hippocampus and OFB, have shown that a significant amount of induced α-syn pathology was found in astrocytes and microglia^13,42^. This suggests that they might be heavily involved in the clearance of α-syn (Fig. 3a,b). Interestingly, in tissue there is clear evidence to suggest that pericytes have macrophage-like properties^43–45^. Pericytes can actively phagocytose and may act as the last line of defence for the BBB, cleaning up the extracellular space and degrading foreign proteins and debris. Altogether, these data suggest that microglia, pericytes and astrocytes may have a role in the uptake and degradation of α-syn.

Additionally, non-neuronal cells may also be involved in the transfer of α-syn from one cell to the next. Primary human brain pericytes have been shown to transfer overexpressed α-syn from one pericyte to another^32^. Through a similar mechanism, it has been demonstrated that astrocytes can transfer aggregated α-syn from one astrocyte to the next^30^. More recently, primary rat astrocytes were shown to accept α-syn inclusions from neurons in culture and efficiently transfer it from astrocyte to astrocyte^27^. However, evidence of these processes occurring *in vivo* is scarce. The fact that both astrocytes and pericytes contain intracellular α-syn in the AON of the human PD OFB suggests that they may have greater involvement in the disease processes. Further work needs to be done to understand whether these cells are actively involved in the transfer or degradation of α-syn in the human brain.

Our study found that oligodendrocytes in the AON did not contain intracellular α-syn. Oligodendrocytes are implicated in multiple system atrophy, which is another synucleinopathy where α-syn pathology is predominantly present in neurons and oligodendrocytes^46^. Previous studies have identified the presence of α-syn in oligodendrocytes in other regions of PD brain ^29,47^. However, the findings in this study showed that oligodendrocytes were spared in the human PD OFB.

PD is largely considered to be a disease of neurons, however, we observe that the α-syn inclusions in non-neuronal cells look similar to the α-syn inclusions seen in neuronal cells in the OFB. We identified that neurons can contain either the small α-syn inclusions, that resemble the types of inclusions seen in the non-neuronal cells (Fig. 2a–c) and the classical large Lewy body like inclusions (Fig. 2e,f). The difference in the size of the inclusions likely pertains to the ability of neurons to sequester α-syn inclusions into large Lewy body like inclusions^48^. The process of Lewy body formation may develop over several stages, starting with diffuse cytoplasmic inclusions that ultimately develop into large Lewy body like inclusions ^48,49^. Another point to consider regarding the size of the α-syn inclusions in the non-neuronal cells is their ability to divide and migrate^50,51^. It is unclear whether this may alter α-syn inclusion size in non-neuronal cells in PD, however, there is evidence to suggest that cells that undergo proliferation may not be affected by aggregated protein inclusions that are found in degenerating mature neurons in diseases such as Huntington’s disease and Machado–Joseph disease^52–54^. Therefore, the difference in α-syn inclusion size could be related to the amount of time that these cells contain the α-syn inclusions and because they are dividing, there is not enough time for Lewy body-like inclusions to develop.

Although we demonstrate that microglia, pericytes and astrocytes contain α-syn in the human PD OFB, we acknowledge a particular caveat. All 11 PD cases used in this study have considerable pathology, suggesting that the AON regions of the OFB have had a prolonged and extensive exposure to α-syn pathology (Fig. 1d,e). Therefore, it is difficult to rule out whether these non-neuronal cells contain α-syn as a consequence of extremely dense α-syn pathology, or whether these non-neuronal cells are actively involved early in the disease processes. Interestingly, *in vivo* studies have demonstrated that injection of α-syn into the OFB in mice leads to a vast number of microglia being affected by α-syn pathology, but only at later time points. At earlier time points, few microglia cells in the OFB were positive for α-syn pathology^13^. This brings into question whether a certain burden of pathology is needed before non-neuronal cells become involved.

This study demonstrates that microglia, pericytes and astrocytes in conjunction with neurons may play an important role in the pathogenesis of PD. Further studies are needed to elucidate the role of non-neuronal cells in the origins and spread of α-syn pathology in the brain. This may provide opportunities for therapeutic intervention by targeting non-neuronal cells.

## Materials and Methods

### Human brain tissue

Post-mortem human OFBs were obtained from the Neurological Foundation Human Brain Bank and the Human Anatomy Laboratory within the Department of Anatomy and Medical Imaging, University of Auckland, New Zealand. Informed consent of the family was obtained prior to autopsy and the University of Auckland Human Participants Ethics Committee approved the protocols (Ref: 011654). All experiments were performed in accordance with relevant guidelines and regulations. The normal cases (n = 11) had no clinical history of neurological disease and no apparent pathological abnormalities upon post-mortem examination. The PD cases (n = 11) had a disease duration ranging from 9–23 years with an average of 16 years (Table 1). Although the post-mortem delay of the normal cases was on average higher than the PD cases, it did not appear to impact the ability to detect phosphorylated α-syn or any of the other markers used in this study (Table 1). Pathological examination by a neuropathologist confirmed the clinical diagnosis of PD by observed presence of Lewy bodies in the substantia nigra as well as pigment incontinence and cell loss in the substantia nigra.

**Table 1:** Human OFB cases used in this study.

Normal cases					
Case number	Age	Sex	Post-mortem delay (hours)	Cause of death	
OFB51	85	M	20	Carbon monoxide poisoning	
OFB55	56	M	35	Myocardial infarction	
OFB57	63	F	36	Effects of diabetes	
OFB58	60	M	36	Asphyxia	
OFB59	67	M	20	Complication of surgery	
H190	72	F	19	Myocardial infarction	
H240	73	M	26.5	Ruptured aneurysm	
H243	77	F	13	Ischaemic heart disease	
H245	63	M	20	Asphyxia	
H246	89	M	17	Myocardial infarction	
H250	93	F	19	Pneumonia	
Average	73 (Range: 56–93)	7:4 (M:F)	24 (Range: 13–36)		
**Parkinson’s disease cases**					
**Case number**	**Age**	**Sex**	**Post-mortem delay (hours)**	**Cause of death**	**Duration of PD (years)**
PD52	84	M	5	Myocardial infarction	12
PD53	79	F	25	Renal failure	9
PD54	78	M	6	Aspiration pneumonia	19
PD56	74	M	10.5	End stage Lewy body disease	12
PD58	82	F	18	—	15
PD63	91	F	5	Parkinson’s disease	22
PD65	67	M	2.25	Parkinson’s disease	9
PD66	73	M	17.5	Aspiration pneumonia	22
PD67	65	M	17	Pneumonia	12
PD77	76	F	6.5	Abdominal carcinoma	23
PD79	77	M	6.5	End stage Lewy body disease	22
Average	77 (Range: 65–84)	7:4 (M:F)	11 (Range: 5–25)		16 (Range: 9–23)

The OFBs were obtained at autopsy and prepared as previously described^11^. Briefly, the OFBs were fixed with 15% formaldehyde in 0.1 M phosphate buffer for 24 hours at room temperature. The OFBs were dehydrated in a graded ethanol series and embedded in paraffin wax using a Leica Tissue Processor. Dehydration comprised of sequential steps from 70%, 80% 2 × 95% and 3 × 100% ethanol for 20 minutes each at room temperature. The tissue was cleared in xylene for 2 × 30 minutes and inserted into molten paraffin wax during three cycles of 25 minutes each. Paraffin blocks with embedded OFBs were sectioned at a thickness of 7 µm on a rotary microtome (Leica Biosystems, RM2235). Sections were mounted individually on Superfrost Plus Slides (Menzel – Gläser) and air-dried for at least 72 hours at room temperature^11^.

Three OFB sections 500 µm apart were selected per case for this study. The first section chosen was a mid-sagittal section of the OFB. The other two sections were then chosen 500 µm either side of the first mid-sagittal section.

### Immunohistochemistry

Slides were heated to 60 °C for 1 hour to melt paraffin wax. Slides were cleared in xylene (2 × 30 mins) and rehydrated in an ethanol series: 2 × 100% (15 mins), 1 × 95% (10 mins), 1 × 80% (10 mins), 1 × 75% (10 mins) followed by 3 × 5 mins in distilled H_2_O. Heat induced antigen retrieval was performed with a Tris – EDTA (pH 9.0) buffer in a pressure cooker (2100 Antigen Retriever, Aptum Biologics Ltd.) for 20 minutes at 121 °C and left to cool for 1.5 hours. Once cooled, slides were washed for 3 × 5 mins in phosphate – buffered saline (PBS) and permeabilized in PBS - T (0.1% Triton X-100 in PBS) for 15 minutes at 4 °C. The sections were then blocked for non-specific secondary antibody binding for 1 hour in 10% normal goat serum (Gibco #16210–072). Primary antibodies (Table 2) were diluted in 1% normal goat serum and incubated on the sections overnight in a humidified chamber. Subsequently slides were washed 3 × 5 mins in PBS. Secondary antibodies (Table 2) were diluted in 1% normal goat serum and incubated on the sections for 3 hours at room temperature. Following this, sections were washed 3 × 5 mins in PBS. Sections were incubated for 5 mins in PBS containing a 1:20 000 dilution of Hoechst 33342 (Molecular probes # H1399) to counterstain nuclei and subsequently washed 3 × 5 mins in PBS. Sections were coverslipped with Prolong Gold (Molecular Probes #P36930). Sections were sealed around the edges of the coverslip using nail polish and stored at 4 °C in the dark until imaged^11^.

**Table 2:** Primary and secondary antibodies used for immunohistochemistry.

Primary Antibodies				
Antibody	Species	Dilution	Catalogue #	Manufacturer
α-synuclein (phospho s129)	Mouse	1:3,000	ab184674	Abcam
α-synuclein (phospho s129)	Rabbit	1:4,000	ab190628	Abcam
PDGFR-β [Y92]	Rabbit	1:200	ab32570	Abcam
GFAP	Chicken	1:4,000	ab4674	Abcam
Iba-1	Chicken	1:250	ab139590	Abcam
NeuN	Chicken	1:500	MABN91	EMD Millipore
PGP9.5	Mouse	1:1,000	ab8189	Abcam
UEA-1	—	1:500	DL-1067	Vector Laboratories
CNPase	Mouse	1:500	sc166558	Santa Cruz Biotechnology
**Secondary Antibodies**				
**Antibody**	**Dilution**	**Catalogue #**	**Manufacturer**	
Goat anti – mouse (488)	1:400	A11029	Thermofisher Scientific	
Goat anti – rabbit (594)	1:400	A11037	Thermofisher Scientific	
Goat anti – chicken (647)	1:400	A21449	Thermofisher Scientific	
Strepavidin – (647)	1:500	S21374	Thermofisher Scientific	

### Imaging and quantification

Sections were imaged using an automated fluorescence microscope; Zeiss Z2 Axioimager equipped with MetaSystems VSlide slide scanner (MetaSystems) running MetaFer (V 3.12.1) coupled with MetaXpress using a 20x magnification objective lens (0.9 NA). Images were stitched using MetaCyte software. Following image capture, the total section scan was viewed using VSViewer (V 1.1.106) (MetaSystems) software. The AON regions were delineated using several antibodies (Fig. 1c). Once the AON regions were identified, this process was applied to sequential sections. Cells with presumed intracellular α-syn were manually counted and marked for their location in the OFB using VSViewer (V 1.1.106) software. All cells with presumed intracellular α-syn were reimaged with a confocal microscope to confirm whether the α-syn was intracellular (Supplementary Fig. 1).

Confocal images were acquired using a FV1000 confocal microscope (Olympus, Japan) with a 40x magnification oil immersion lens (1.00 NA), 60 x magnification oil immersion lens (1.35 NA) or 100 x magnification oil immersion lens (1.40 NA) in a Z-series using a step size of 0.5 µm. Orthogonal projections with maximum intensity Z-projections were generated using ImageJ software.

### Manual cell counts in the AON and calculation of the area fraction of α-synuclein (cell types)

To count the total number of each cell type in the AON of the OFBs a manual counting method was used. Briefly, the AON regions were extracted using the VSViewer software and opened in ImageJ. Background intensity was measured using a 100 µm^2^ box over three areas and averaged. The multi-point tool was used to pinpoint the brightest part of cellular labelling. The value of the integrated intensity of the point was recorded, background intensity was subtracted and if the value of the integrated intensity of the cell was above a determined threshold for that marker, it would be counted as a cell.

The total area fraction of α-syn in the OFB was determined by thresholding the α-syn labelling in the AON or across the entire OFB and tract. The thresholded area was subsequently normalised to total tissue area of the OFB or AON regions to obtain the percentage of α-syn coverage in a given area.

### Statistical analysis

In general, data are presented as mean ± standard deviation (SD) from the average of three different sections per case. Data visualization and statistical hypothesis testing was performed using GraphPad Prism Version 8.02. Linear regression was used to analyse correlations. One-way analysis of variance (ANOVA) was used when comparing across cell types with Tukey’s multiple comparison adjustment and unpaired t-tests were used when comparing the area fraction of α-syn inside versus outside of the AON. Statistical significance was set as p < 0.05

## Supplementary information

Supplementary Information.

Supplementary Figure.

## Data Availability

The datasets generated during and/or analysed during the current study are available from the corresponding author on reasonable request.
