# Supplementary material for: α-synuclein inclusions are abundant in non-neuronal cells in the anterior olfactory nucleus of the Parkinson’s disease olfactory bulb
Source: Sci Rep. 2020 Apr 21;10:6682. doi: 10.1038/s41598-020-63412-x (PMC7174302; doi:10.1038/s41598-020-63412-x)
Supplement: Supplementary file 1 — Supplementary Information. [file 41598_2020_63412_MOESM1_ESM.docx]

α-synuclein inclusions are abundant in non-neuronal cells in the anterior olfactory nucleus of the Parkinson’s disease olfactory bulb

Taylor J. Stevenson^1,2^, Helen C. Murray^1,2,3^, Clinton Turner^2,4^, Richard L. M. Faull^1,2^, Birger V. Dieriks^1,2^*, Maurice A. Curtis^1,2^*

Affiliations:

^1^ Department of Anatomy and Medical Imaging, University of Auckland, Auckland, New Zealand

^2^ Centre for Brain Research, University of Auckland, Auckland, New Zealand

^3^ Laboratory of Functional and Molecular Imaging, National Institute of Neurological Disorders and Stroke, National Institutes of Health, Bethesda, MD 20892, USA

^4^Deparment of Anatomical Pathology, LabPlus, Auckland City Hospital, Auckland, New Zealand

*Equal contribution for last author

Corresponding author: Professor Maurice Curtis

E-mail: [m.curtis@auckland.ac.nz](mailto:m.curtis@auckland.ac.nz)

Telephone: +64 9 923 6999


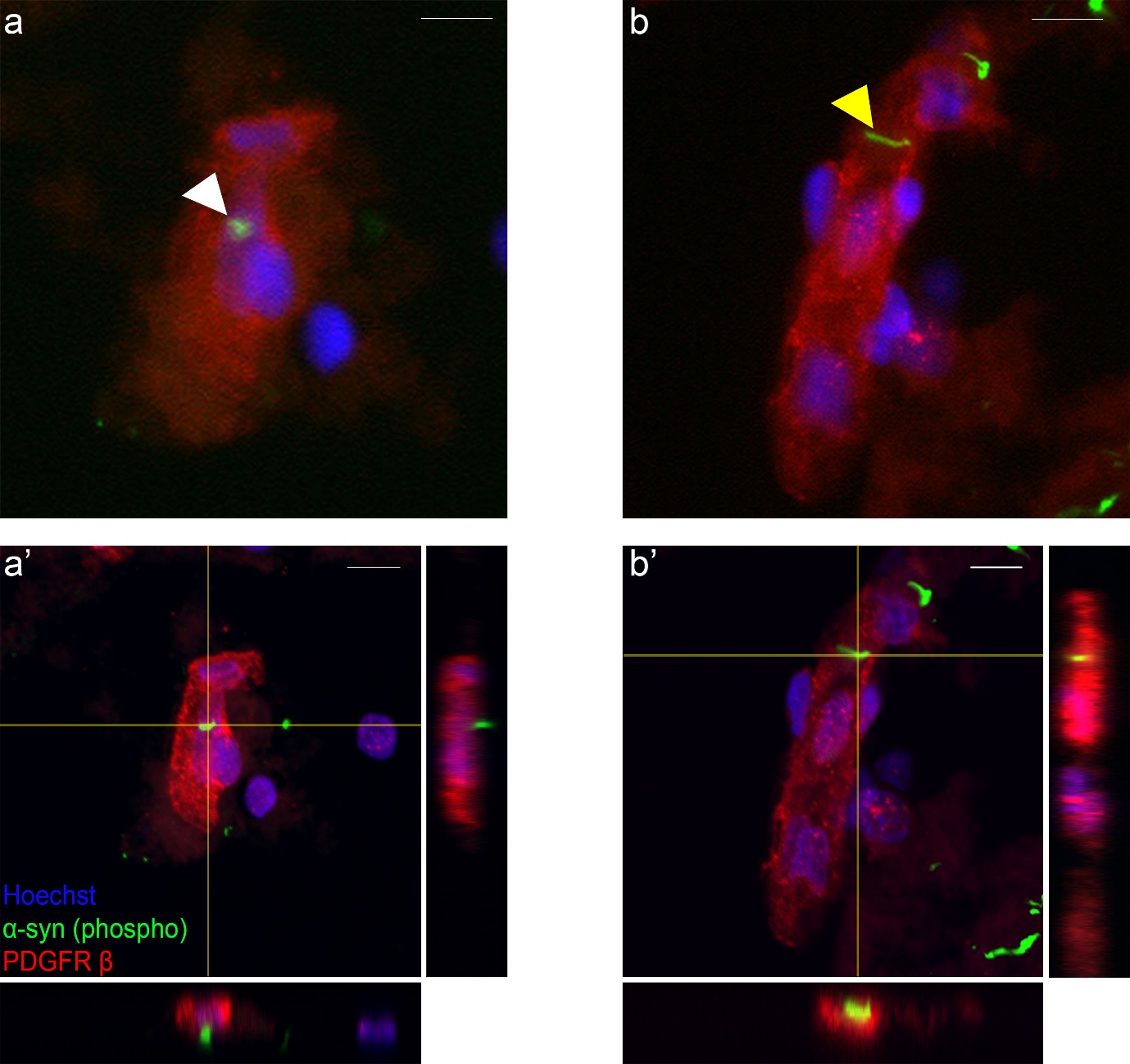


**Supplementary Figure 1** Methodology for identifying cells with intracellular α-syn. (a, b) Cells with presumed intracellular α-syn were first identified using a slide scanning microscope (VSViewer V 1.1.106 - https://metasystems-international.com/en/products/solutions/tissue-imaging/). Once identified, the same cells were reimaged with confocal microscopy to confirm whether α-syn is (a’) extracellular or (b’) intracellular. Yellow arrow indicates α-syn inclusions that are intracellular. White arrow indicates α-syn inclusions that are extracellular. Scale bar, 10 µm.
