# Supplementary figures and images for: α-synuclein inclusions are abundant in non-neuronal cells in the anterior olfactory nucleus of the Parkinson’s disease olfactory bulb
Source: Sci Rep. 2020 Apr 21;10:6682. doi: 10.1038/s41598-020-63412-x (PMC7174302; doi:10.1038/s41598-020-63412-x)

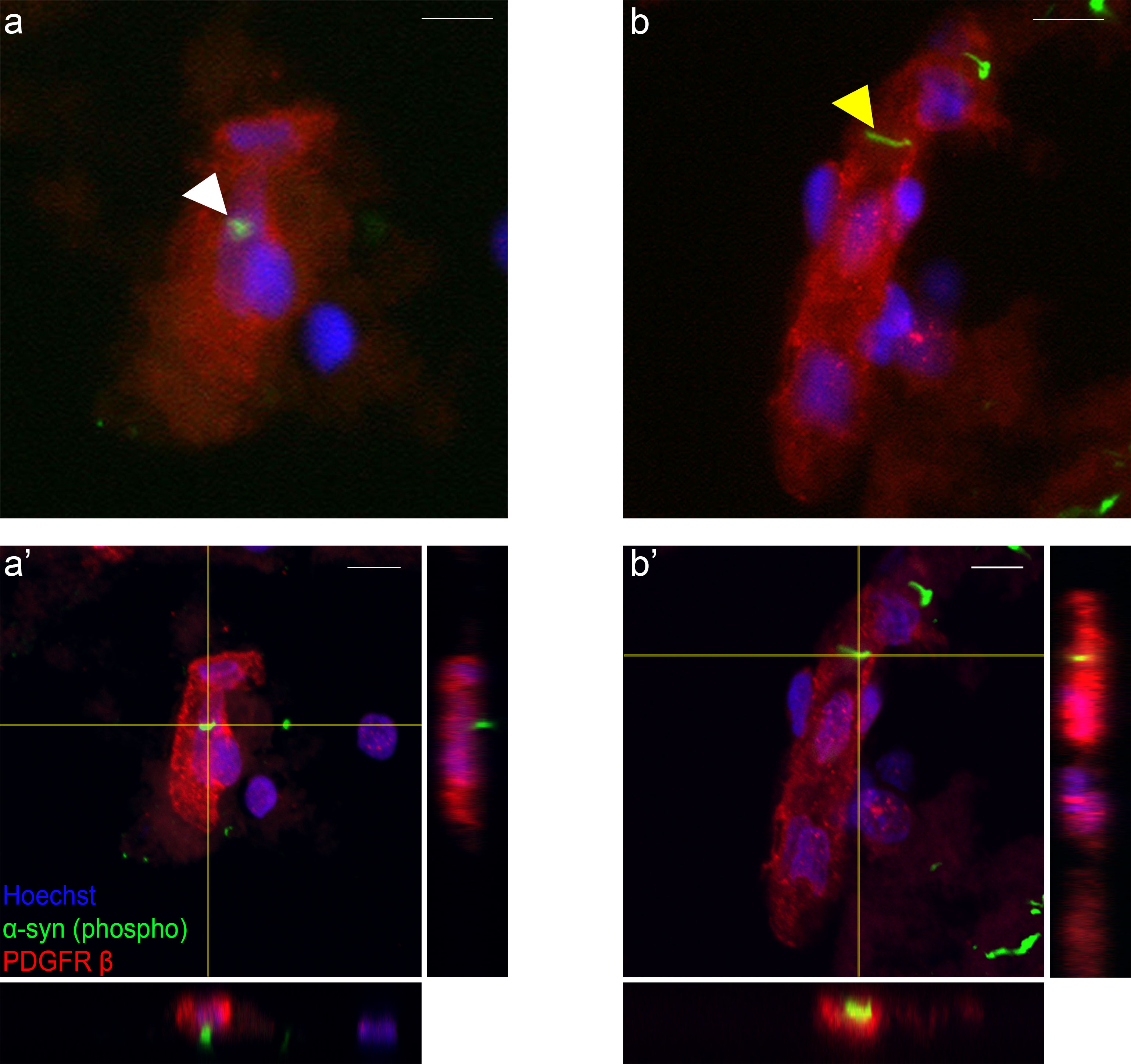

Supplement: Supplementary file 2 — Supplementary Figure. [file 41598_2020_63412_MOESM2_ESM.jpg]
